# Characterization of the effect of sample quality on high density oligonucleotide microarray data using progressively degraded rat liver RNA

**DOI:** 10.1186/1472-6750-7-57

**Published:** 2007-09-13

**Authors:** Karol L Thompson, P Scott Pine, Barry A Rosenzweig, Yaron Turpaz, Jacques Retief

**Affiliations:** 1 Center for Drug Evaluation and Research, US Food and Drug Administration, Silver Spring, MD, USA; 2 Affymetrix Inc., Santa Clara, CA, USA; 3 Current- Illumina Inc., San Diego, CA, USA

## Abstract

**Background:**

The interpretability of microarray data can be affected by sample quality. To systematically explore how RNA quality affects microarray assay performance, a set of rat liver RNA samples with a progressive change in RNA integrity was generated by thawing frozen tissue or by *ex vivo *incubation of fresh tissue over a time course.

**Results:**

Incubation of tissue at 37°C for several hours had little effect on RNA integrity, but did induce changes in the transcript levels of stress response genes and immune cell markers. In contrast, thawing of tissue led to a rapid loss of RNA integrity. Probe sets identified as most sensitive to RNA degradation tended to be located more than 1000 nucleotides upstream of their transcription termini, similar to the positioning of control probe sets used to assess sample quality on Affymetrix GeneChip^® ^arrays. Samples with RNA integrity numbers less than or equal to 7 showed a significant increase in false positives relative to undegraded liver RNA and a reduction in the detection of true positives among probe sets most sensitive to sample integrity for *in silico *modeled changes of 1.5-, 2-, and 4-fold.

**Conclusion:**

Although moderate levels of RNA degradation are tolerated by microarrays with 3'-biased probe selection designs, in this study we identify a threshold beyond which decreased specificity and sensitivity can be observed that closely correlates with average target length. These results highlight the value of annotating microarray data with metrics that capture important aspects of sample quality.

## Background

It is recommended that the highest quality RNA be used for genomic analyses. However, in some cases, such as human autopsy samples or paraffin embedded tissues, high quality RNA samples may not be available. It is therefore important to understand how RNA quality affects the interpretation of the results and also how reliable current quality measures are at indicating RNA quality issues. It has been reported that gene expression profiling on Affymetrix GeneChip arrays is relatively tolerant to moderate RNA degradation and to the 5'-truncation that occurs during the two successive rounds of *in vitro *transcription needed to detect small sample quantities [1-3]. Some samples fall within a "grey zone" of sample quality, where there is some loss of RNA integrity but the samples still pass RNA quality thresholds. It is unknown how differences in RNA integrity within the "grey zone" affect the data interpretation. More information is needed to help guide the generation of best practice recommendations for sample handling and the evaluation of the quality of genomic studies submitted to public databases to fulfill journal requirements and to regulatory agencies.

The recommended method for preparing target from RNA for hybridization to Affymetrix microarrays is based on the Eberwine procedure [4]. The sample labeling and amplification method starts with cDNA synthesis from the polyadenylation (polyA) site followed by the generation of cRNA from the sense strand of the cDNA via an incorporated T7 primer sequence. Because this process generates labeled target with a 3' bias, Affymetrix GeneChip Rat Expression Set 230 (RAE230A) arrays are designed to contain probes that reside within the 600 nucleotides (nt) most proximal to the 3' end of each transcript [5]. Where alternative polyA sites are identified within 600 nt of each other, the probe selection region is based on the most upstream site. The housekeeping genes beta-actin (*Actb*) and glyceraldehyde-3-phosphate dehydrogenase (*Gapdh*) serve as internal controls of RNA quality and the target preparation process. Probe sets have been designed to hybridize to the 5', middle (M), or 3'-regions of these control transcripts. High signal ratios of the 3' probe set to the 5' probe set are indicative of either RNA degradation or target synthesis problems. It has been recommended that samples should have a 3'/5' signal ratio for *Gapdh *of no more than 3 [6].

Various methods for measuring sample quality pre- and post-hybridization have been proposed [7-10]. In this study the degree of RNA degradation was standardized by use of an Agilent 2100 Bioanalyzer to assign an RNA integrity number (RIN) to each sample. The RIN software algorithm classifies the integrity of eukaryotic total RNA on a scale of 1 to 10 (most to least degraded) based on the most informative features of an electropherogram of the 18s and 28s rRNA peaks [11].

In this study a set of rat liver samples with a progressive loss in RNA quality was generated. This dataset was used to characterize individual probe set sensitivity to RNA degradation and to evaluate the effect of RNA integrity on the sensitivity and specificity of microarray data generated on Affymetrix GeneChip arrays.

## Results

### Effect of sample handling on RNA integrity

**Figure 1:**
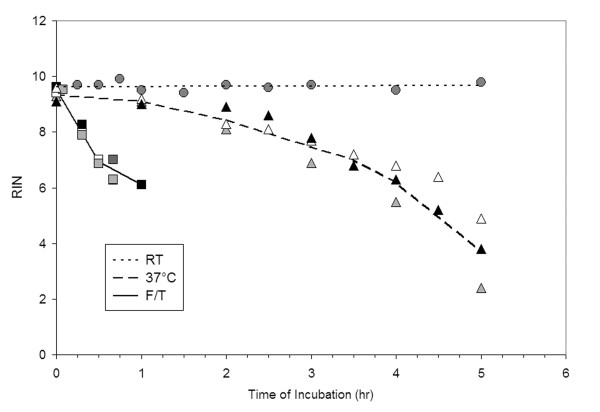
**Time course of RNA degradation induced by different tissue handling conditions**. RNA was prepared from liver sections incubated at room temperature (RT) (circles), 37°C (triangles), or frozen and thawed at room temperature (F/T) (squares). The dotted line represents the linear trendline for the RT incubated sample set. Dashed or solid lines connect the mean values at hourly or semi-hourly time points for the 37°C or F/T handling condition sets, respectively. Symbol shading indicates replicate experiments conducted on different days from independent sources of tissue.

The methods used to harvest and preserve source tissue for gene expression analyses can impact the quality of isolated mRNA and the reliability of microarray data generated from this source. We investigated the relative impact of several different tissue handling conditions on RNA integrity. These conditions were designed to model the effect of time between necropsy or sacrifice and sample processing (incubation at room temperature or 37°C) or between removal from storage and sample processing (time of thaw of frozen sample). Fresh liver tissue was incubated up to 6 hr at room temperature without a measurable effect on RNA integrity, as measured by RIN (Figure 1). RNA in fresh liver tissue proved to be remarkably stable. RNA degradation was only observed after fresh liver tissue was incubated at 37°C for 120 min or more and poor quality RNA (RIN ≤ 7) appeared after 3.5 hours of incubation at 37°C. RNA degradation was much more rapid in frozen tissue. Poor quality RNA (RIN ≤ 7) was isolated from frozen tissue thawed for 15–30 min at room temperature.

### Sample characterization by RNA quality metrics

**Figure 2:**
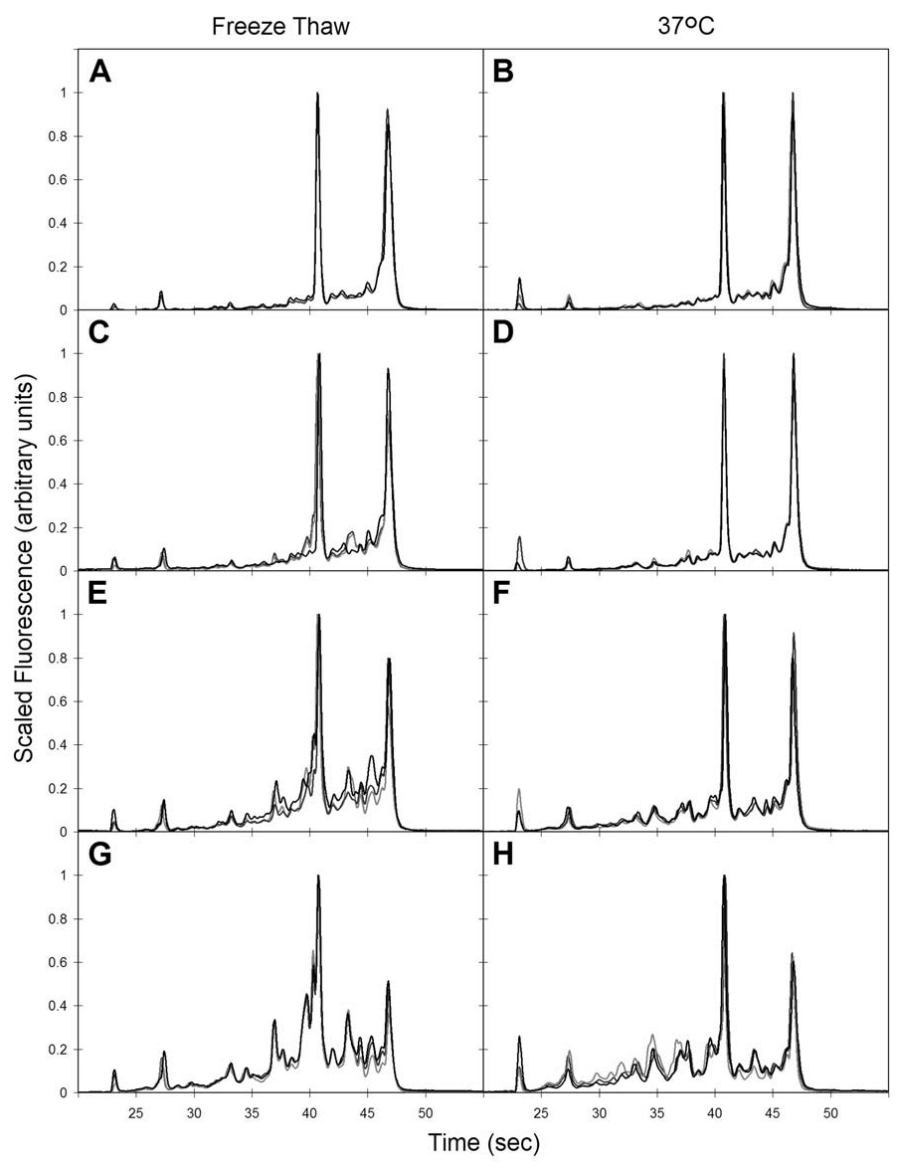
**Electrophoretic tracings of RNA progressively degraded by different handling methods**. Rescaled tracings were overlaid from three RNA samples from independent experiments with similar RIN values. Tracings for RNA from samples degraded by freeze/thaw (A, C, E, G) or by 37°C incubation (B, D, F, H) and with values of RIN 9.5 (A and B), RIN 8 (C and D), RIN 7 (E and F), or RIN 6 (G and H) are shown.

For each of the sample handling conditions that induced RNA degradation (37°C incubation or freeze/thaw (F/T)), sets of progressively degraded RNA were generated in independent experiments, with each experiment using a single liver lobe from a different individual animal as source tissue. Each of these sets contains a minimum of 12 RNA samples across three or more replicate experiments with RIN values ranging from 9.5 to 5. Electrophoretic tracings of RNA with RIN measurements of 9.5, 8, 7, or 6 are shown in Figure 2 for three independent samples in each sample handling set. The tracings are highly similar between samples with the same RIN, but show subtle differences between the two handling methods for a given RIN value. Within each handling condition, 3 samples with similar RIN values were grouped as replicate samples in all additional analyses on the effects of RNA degradation.

Next, the relationship was examined between RIN and 3 other RNA quality metrics (28s/18s rRNA ratio, cRNA yield, and cRNA length) that are generated before or during microarray sample preparation (Table 1). Of these 3 metrics, RIN value was most highly correlated with average cRNA length across a set of 29 samples generated from both handling methods (*r *= 0.86). RIN values also correlated fairly well with cRNA yield and with 28s/18s ribosomal RNA ratios (*r *= 0.84 and *r *= 0.82, respectively). Some differences in the correspondence between RIN value and the other 3 RNA metrics were observed between the two different handling methods. In general, there was a stronger decreasing trend in metric value as a function of RIN for RNA degraded by F/T than for RNA degraded by 37°C incubation.

**Table 1:** RNA metrics associated with RIN value and RNA degradation method. Average values and standard deviations are reported for metrics associated with samples in each RIN class (n = 3).

***Handling condition***	***RIN class***	***RIN***	***28s/18s rRNA***	***cRNA yield***	***cRNA length***	***3'/5' GAPDH***	***3'/5' β-actin***
F/T	9.5	9.5 ± 0.1	1.43 ± 0.15	86 ± 8	1923 ± 157	1.17 ± 0.06	1.70 ± 0.35
	8	8.1 ± 0.2	1.00 ± 0.00^a^	63 ± 4^b^	1398 ± 116^a^	1.77 ± 0.21	1.93 ± 0.06
	7	7.0 ± 0.1	0.70 ± 0.10^a^	44 ± 12^a^	1136 ± 207^a^	3.05 ± 0.95^b^	3.62 ± 1.18^b^
	6	6.2 ± 0.1	0.50 ± 0.10^a^	38 ± 3^a^	878 ± 62^a^	5.50 ± 0.8^a^	5.27 ± 0.86^a^

37°C	9.5	9.5 ± 0.2	1.35 ± 0.13	74 ± 16	2294 ± 236	1.01 ± 0.02	1.34 ± 0.02
	9	9.1 ± 0.1	1.33 ± 0.06	83 ± 7	2110 ± 322	1.24 ± 0.20	1.49 ± 0.26
	8	8.2 ± 0.1	1.33 ± 0.06	61 ± 19	1834 ± 293	1.29 ± 0.18	1.60 ± 0.04
	7	7.0 ± 0.2	1.07 ± 0.15^b^	42 ± 6	1210 ± 158^a^	2.00 ± 0.21^a^	2.12 ± 0.20^a^
	6	6.1 ± 0.5	0.90 ± 0.10^a^	39 ± 18^a^	1244 ± 223^a^	1.96 ± 0.41^a^	2.29 ± 0.40^a^

^a^P < 0.01 compared to RIN 9.5 set

^b^P < 0.05 compared to RIN 9.5 set

### Microarray quality metrics and signal changes induced by RNA degradation

To systematically assess the effect of degree of RNA integrity on microarray data, RNA samples in each RIN group were analyzed on Affymetrix GeneChip Rat Expression 230A arrays for both handling conditions. Most of the global microarray quality metrics that are summarized in Affymetrix report files were within normal ranges for the 27 samples with RIN ≥ 6. All samples had percent present calls within 10% of the mean value (49%). The scale factors (SF) for all but one hybridization were within 2 SD of the mean (SF 1–3) and all were within 3 SD of the mean. All samples except one RIN 7 sample and 3 RIN 6 samples in the F/T set had 3'/5' *Gapdh *ratios below the recommended threshold of 3. The effect on 3'/5' *Gapdh *and 3'/5' *Actb *ratios corresponded well with average cRNA transcript length (Table 1). The 3' boundaries of the target sequences (TargetSeq) for the 5' *Gapdh *and *Actb *probe sets are located about 883 and 855 nt, respectively, upstream of the 3' end of their corresponding RefSeqs and span about 1150 nt in length [see Additional file 1]. Targets that are less than 878 nt in length on average (i.e. the F/T RIN 6 samples) would be expected to exhibit significantly reduced hybridization to these probe sets.

**Figure 3:**
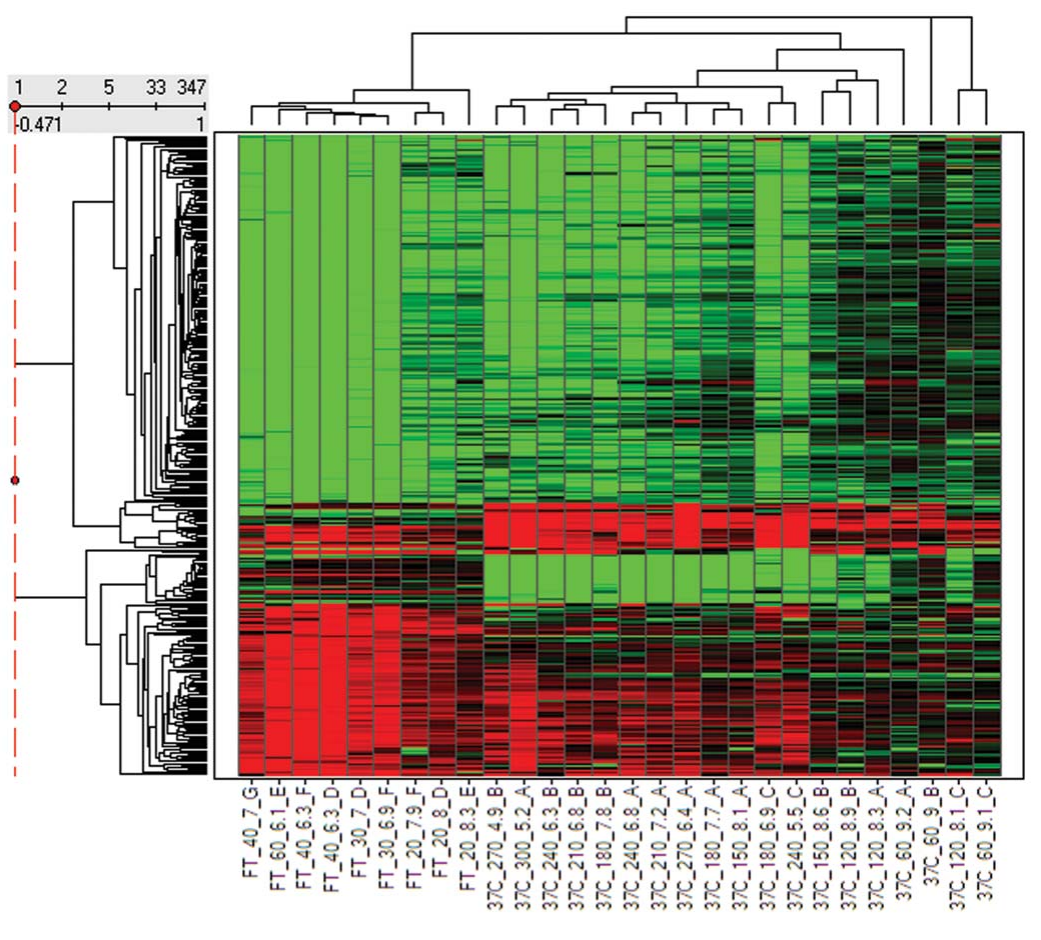
**Hierarchical clustering of significantly changed genes from samples progressively degraded by different tissue handling conditions**. Sample labels are concatenated from handling condition, time of incubation, RIN, and study code. Log_2 _ratios calculated for each sample relative to the average of three zero-time controls are mapped on a green (-1) to red (1) color scale.

The quality metrics discussed so far are summary metrics that provide an assessment or surrogate measure of the overall integrity of the sample. Individual probe set signals may vary in their sensitivity to RNA degradation. To visualize the effect of handling condition and degree of RNA degradation on individual gene expression profiles, we limited the comparison set to the genes that were most affected by sample incubation (347 probe sets that were changed by 2-fold or greater in at least 30% of all non-control F/T or 37°C samples compared to zero time controls). The relationship between the log_2 _ratio data for the filtered sets of noncontrol samples from the F/T and 37°C incubations was displayed by plotting the heatmap and dendrograms resulting from average linkage hierarchical clustering (Figure 3). The samples clustered primarily by handling condition and then by degree of degradation. In general, four different patterns of probe set responses are visualized in the heat map. The majority of probe sets showed a decrease in signal induced by degradation that was independent of handling condition and observable in even moderately degraded (RIN 8) samples. A second, smaller cluster exhibited an increase in signal induced by degradation by either method. The expression levels of a third subset of genes were selectively altered by *ex vivo *incubation at 37°C [see Additional File 2]. The 10 genes in this cluster are primarily involved in cellular defense responses like the mitogen activated protein kinase (MAPK) pathway (*Dusp1*, *Hspa1a*, *c-Jun*), immune response (*Cxcl1*), response to hypoxia (*Egr-1*) [12], cell growth regulation (*Btg2*, *Myd116*, *Bhlhb2*) or other stress responses (*Zpf36, Slc25a25*). Over expression of *Zfp36*, *Btg2*, *c-Jun*, and *Egr-1 *has also been reported to occur in surgically extirpated prostate tissue after 1 hr of warm ischemia [13]. *Dusp1 *and *Egr-1 *are also 2 of 14 gene transcripts that increased in peripheral blood mononuclear cells prepared by Ficoll-Hypaque density centrifugation at 21°C compared to 8°C [14]. Independent confirmation of an increase in *Egr1 *mRNA levels (5-fold after 1 and 3 hr incubation at 37°C) was conducted using qRT-PCR (data not shown). A fourth cluster of 25 probe sets were selectively decreased in signal after 2 hr *ex vivo *incubation at 37°C. More than 75% of these probe sets hybridize to transcripts that are either highly expressed in immune cells or involved in immune function [see Additional file 2]. *Ex vivo *incubation may have caused a selective loss in the presence, function or integrity of immune cells in the liver samples.

### Characterization of probe set sensitivity to RNA degradation

The correlation between RNA degradation by F/T and average cRNA length observed in Table 1 suggests that relative probe set position on a target reference sequence may be a determinant of sensitivity to degradation. To examine this further, probe sets were first identified that showed a statistically significant difference in signal level between RIN groups and a progressively increasing or decreasing trend in average signal between RIN 9.5, 8, 7, and 6 sets generated by F/T (INC or DEC, respectively). Only probe sets that mapped to a single reference sequence (RefSeq) transcript containing a terminal polyA sequence ≥ 10 nt were selected in order to accurately measure probe set location relative to the reverse transcription initiation site. Using these criteria, 89 DEC and 12 INC probe sets were identified. 61 probe sets were also identified that were relatively invariant in signal as a function of RIN (INV).

**Figure 4:**
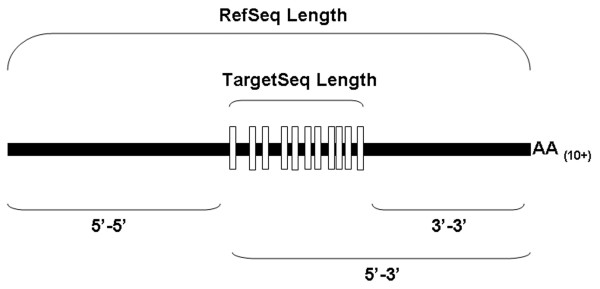
**Probe set distance metrics**. Illustration of distance metrics for a hypothetical probe set (vertical open bars) in relation to the 5'- and 3'-termini of the corresponding RefSeq (solid horizontal bar).

For probe sets classified as INV or DEC, seven measurements were made to characterize the location and length of each probe set target sequence on its corresponding reference transcript sequence (Figure 4). The INC set was not further characterized because of the small sample size. The metrics were RefSeq length, TargetSeq length, TargetSeq length/RefSeq length, 5'-3' distance (distance from 5' end of the TargetSeq to the 3' end of the RefSeq), 3'-3' distance (distance from 3' end of the TargetSeq to the 3' end of the RefSeq), 5'-5' distance (distance from 5' end of the RefSeq to the 5' end of the TargetSeq), and average RIN 9.5 signal. A mean and standard deviation for each distance metric was calculated within DEC or INV groups (Table 2). Individual measurements for each probe set in the DEC and INV groups are tabulated in Additional file 3.

**Table 2:** Distance metrics associated with probe set sensitivity to F/T RNA degradation. Probe sets were classified as invariant (INV) to degradation or as decreasing in signal (DEC) in response to degradation. DEC probe sets were further divided into two classes based on probe set location relative to the corresponding reference sequence termini.

***Distance metric***	***INV***	***DEC***	***DEC (5'-3' < 1000)***	***DEC (5'-3' > 1000)***
5'-3' distance	638 ± 386	1381 ± 665^a^	646 ± 171	1637 ± 576^b^
3'-3' distance	211 ± 388	973 ± 653^a^	289 ± 228	1212 ± 579^b^
5'-5' distance	1072 ± 783	904 ± 764	1250 ± 874	784 ± 689
RefSeq length	1711 ± 845	2285 ± 901^a^	1896 ± 830	2420 ± 891^b^
TargetSeq length	479 ± 72	451 ± 109	406 ± 111^c^	467 ± 105
TargetSeq/RefSeq	0.37 ± 0.21	0.22 ± 0.10^a^	0.25 ± 0.14^c^	0.21 ± 0.08^b^
Avg log_2 _RIN 9.5 signal	11.5 ± 1.3	10.0 ± 1.7^a^	10.0 ± 1.7^c^	10.1 ± 1.8^b^

Count	61	89	23	66

^a^P < 0.0001 in unpaired two-tailed t-test comparisons of INV and DEC sets

^b^P < 0.001 in a Tukey's post-test comparison of a one-way ANOVA

^c^P < 0.01 in a Tukey's post-test comparison of a one-way ANOVA

The correlation between each distance metric and probe set sensitivity to RNA integrity was examined in an unpaired t-test comparison of DEC and INV probe set metrics. Probe sets that decreased in signal as a function of decreasing RNA integrity tended to be located significantly farther from the 3' end of their target transcript sequences than INV probe sets (P < 0.0001) (Table 2). While average TargetSeq length did not significantly differ between DEC and INV groups, DEC probes tended to map to longer RefSeq transcripts and therefore had lower TargetSeq Length/RefSeq Length ratios. DEC probe sets also tended to be lower in signal in undegraded (RIN 9.5) samples than INV probe sets.

**Figure 5:**
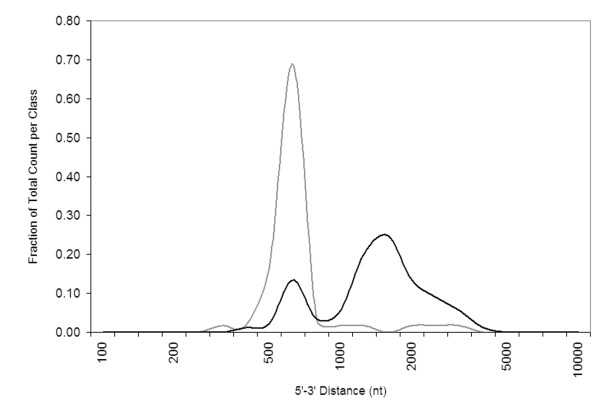
**Histogram of 5'-3' distances for probe sets with different responses to RNA degradation**. 5'-3' distances for probe sets that are INV (grey) or DEC (black) are plotted as a fraction of the total number in each set.

Unlike INV probe sets, the 5'-3' distances of DEC probe sets were bimodally distributed with maxima near 650 and 1600 nt (Figure 5). The DEC probe sets were divided into two groups with 5'-3' distances either less than or greater than 1000 nt and analyzed further. The majority (66/89) of DEC probe sets including AFFX_Rat_GAPDH_5_at and AFFX_Rat_beta-actin_5_at had 5'-3' distances > 1000 nt. All metrics that were significantly different for the DEC group as a whole were also significantly changed for this subset. The 23 DEC probe sets with 5'-3' distances < 1000 nt (which includes AFFX_Rat_beta-actin_M_at) were significantly different from INV probe sets in TargetSeq length, TargetSeq Length/RefSeq Length, and average RIN 9.5 signal (Table 2).

### Effect of level of RNA integrity on microarray performance

Sensitivity (the rate of detection of true positives among all positives) and specificity (the rate of detection of true negatives among all negatives) are important performance objectives for microarray experiments. In toxicogenomic experiments that are designed to measure the effect of time and dose level of treatment on gene expression, misleading results can be generated by confounding variables such as RNA degradation, tissue sectioning, diurnal effects, etc. The effect of RNA degradation on assay specificity was measured by comparing control liver samples in which RIN level was the independent variable. Statistical Analysis of Microarrays (SAM) was applied at a median false discovery rate (FDR) of 0.1 in two-sample comparisons of undegraded control liver RNA (RIN 9.5) with liver samples of decreasing RIN value generated by F/T. Comparisons of RIN 9.5 samples with RIN 8 or 7 samples yielded 16 or 255 false positives, respectively, using signals derived with either MAS5 or PLIER. A high number of statistically significant changes in signal level were observed for RIN 9.5 and RIN 6 sample comparisons (9243 using PLIER and 4203 using MAS5).

The effect of RIN level on sensitivity was assessed using receiver operating characteristic (ROC) plots that measure diagnostic accuracy. ROC plots are generated by plotting sensitivity (true positive fraction) versus 1-specificity (false positive fraction) along a continuum of decision thresholds (P-value cutoffs). Known gene expression changes were modeled *in silico *using a mixed tissue paradigm designed to measure microarray performance [15]. Two mixtures composed of different proportions of rat testis, brain, liver, and kidney RNA are the components of a reference material that has signal ratios of 1:4, 2:1, 3:2, and 1:1 in tissue-selective probe sets. This mixed tissue RNA design can be effectively modeled *in silico *from array data for each tissue RNA component in the mixture. Microarray signal data from rat liver RNA with different levels of RNA integrity (RIN 9.5, 8, 7, or 6) generated by F/T, was combined *in silico *with signal data from rat brain, testis, and kidney RNA. Assay sensitivity for detecting a true positive fold change of 1.5-, 2-, or 4-fold at a fixed false positive rate of 10% was calculated for each RNA quality level from data modeled with proportional differences in liver-selective (LS) signal and a 1:1 ratio of kidney-selective (KS) signal. These calculations were made using the entire set of 292 LS probe sets unselected for sensitivity to RNA degradation (LS_ALL) or subsets of probe sets that either significantly decrease (LS_DEC) or are invariant in signal (LS_INV) as a function of RNA quality as true positives and 188 KS probe sets as true negatives (Table 3).

**Table 3:** Effect of RIN and probe sensitivity to RNA degradation on assay sensitivity. The percent sensitivity for a false positive rate of 10% [and 95% Confidence Interval] is reported for liver selective probe sets that significantly decrease in response to RNA degradation (LS_DEC), all liver selective probe sets (LS_ALL), and liver selective probe sets that are invariant to RNA degradation (LS_INV).

Fold change	RIN group	LS_DEC	LS_ALL	LS_INV
1.5	9.5	97.2 [85.8, 99.7]	92.9 [88.2, 96.1]	100 [94.0, 100]
	8	92.4 [78.7, 98.1]	92.0 [87.5, 95.2]	100 [93.6, 100]
	7	72.7 [55.2, 85.9]	88.7 [82.0, 93.4]	100 [90.3, 100]
	6	68.4 [51, 82.5]	88.3 [82.9, 92.4]	100 [84.5, 100]
2	9.5	99.2 [90.7, 100]	96.3 [93.3, 98.1]	100^a^
	8	94.3 [82.6, 98.7]	94.7 [91.6, 96.8]	99.9 [80.3, 100]
	7	84.2 [68.7, 93.6]	93.8 [89.4, 96.7]	100 [39.5, 100]
	6	83.6 [68.3, 93.1]	93.3 [89.3, 96.0]	100 [79.7, 100]
4	9.5	99.0 [89.3, 100]	96.9 [94.5, 98.4]	100^a^
	8	96.4 [85.8, 99.4]	95.7 [93.1, 97.5]	100^a^
	7	87.8 [74.1, 95.4]	95.3^b^	100^a^
	6	87.6 [74.2, 95.2]	94.6 [91.6, 96.7]	100^a^

^a^No CI (for perfect discrimination, data cannot be fitted to a ROC curve)

^b^Value interpolated from empirical data using Weibull cumulative discrimination function

Assay sensitivity was markedly decreased by the use of LS_DEC or LS_ALL probe sets as analytes for detecting modeled changes of 1.5-, 2-, and 4-fold as a function of RIN level. The effect was most pronounced for LS_DEC probes, for 1.5-fold change detection, and for RNA quality of RIN ≤ 7. ROC plots that used LS_INV probe sets as analytes showed no change in sensitivity as a function of either RIN level or fold change detection.

## Discussion

In this study, it was observed that time after thawing had a greater effect on RNA integrity than time of incubation of liver tissue after surgical removal at either room temperature or 37°C (Figure 1). Freezing disrupts tissue structure, rendering the tissue highly sensitive to RNA degradation. In contrast, autolysis of fresh liver tissue appeared to be a much slower process. To minimize the potential impact of RNA degradation on microarray data, resected tissue should be sectioned and either flash frozen or immersed in a tissue stabilization solution such as RNALater. Archived frozen tissue should be quickly disrupted and homogenized in denaturing solutions after removal from storage. Homogenization can be performed more rapidly if tissue is cut into smaller sections prior to freezing.

Although *ex vivo *incubation of tissue for several hours had little effect on RNA integrity, it did induce changes in the expression of ischemia-induced and early immediate genes, as has been reported by others [13,16,17]. Many inflammatory response gene transcripts are inherently unstable as a mechanism to control cellular response to certain stimuli [18]. The increases in signal observed with 37°C incubation could result from *de novo *transcription or stabilization of labile mRNAs through, for example, the activation of MAPK or other signaling pathways [19]. Incubation of liver sections at 37°C also induced a selective decrease in a set of genes associated with immune function. Delay in sample processing has been observed to cause a decrease in the levels of selective transcripts in blood cells [16]. Alternatively, this result could have arisen from selective loss of an immune cell population in liver through diffusion or autolysis.

A majority (≥ 75%) of the probe set signals identified as most sensitive to sample incubation at 37°C or after thawing (see Figure 3) are expressed in whole tissue RNA preparations from brain, kidney, and heart in addition to liver (data not shown). Although differences in the kinetics of postmortem RNA degradation have been observed between tissue types [20], it is anticipated that signals for most of these 347 probe sets would also be sensitive to sample integrity in these other tissues. Similarly designed studies using other tissues may identify additional probe sets that are sensitive to sample integrity but not expressed in rat liver.

A subtle but reproducible difference in the relationship between RIN value and qualitative (electrophoretic tracings) or other quantitative RNA metrics (28s/18s rRNA ratio, cRNA yield, and cRNA length) was observed between samples generated by different tissue handling methods (Table 1 and Figure 2). The method of degrading RNA may release or activate ribonucleases with different specificities or differentially affect ribonuclease access to substrate. For example, ribonucleases 1 and 4 have different pH optima and substrate preferences for poly(C) and poly(U) [21]. Freeze/thaw may be the more relevant method of inadvertent sample degradation associated with toxicogenomic studies. The correlations observed here between RIN levels generated by F/T of liver tissue and other sample quality metrics may not necessarily be applicable for other mechanisms of RNA degradation (e.g., the introduction of exogenous RNase during handling) or for other tissues. For example, a threshold RIN value of 7.8 has been recently proposed for optimal RNA reliability for analysis of human tumor samples on Affymetrix GeneChip arrays, where reliability was defined as a 3'/5' *Gapdh *ratio threshold ≤ 1.25 [10]. In our study, only samples with RIN ≥ 9 had 3'/5' *Gapdh *ratios ≤ 1.25.

More interlaboratory studies are needed to evaluate the reproducibility of RIN and its correlation to performance on multiple array formats before a RIN threshold can be recommended as a component of best practices for microarray data generation. The advantage to RIN as a metric is that it is an automated measurement made prior to performing expensive *in vitro *transcription (IVT) assays and array hybridizations. Although average cRNA length correlated well with microarray sample quality in this study, this value is currently not an automated measurement that is calculated by Agilent Bioanalyzer software and needs to be estimated from electropherograms by the end user.

The primary effect of RNA degradation on samples analyzed on microarrays was a decrease in the average length of products that are reverse transcribed and amplified using T7 polymerase. The multiple rounds of *in vitro *transcription that are used to generate samples from small amounts of RNA from biopsies or laser-captured microdissections also induce a decrease in transcript cRNA yield and length [1]. Amplifying target from small samples was associated with a loss of signal for gene transcripts with high GC content and with a greater number and length of predicted hairpin formations [22]. We did not observed any difference in GC content between probe sets that were most and least sensitive to RNA degradation generated by F/T in rat liver (data not shown).

A relatively small percentage (~4%) of probe sets that are on RAE230A arrays and expressed in liver were found to be similar in sensitivity to cRNA target length as the 5'-probe sets for *Gapdh *or *Actb*. Of the probe sets with this sensitivity that also had verifiable transcription termini, most (~75%) were located more than 600 nt upstream of the 3'-end of their target sequences. For the remaining DEC probe sets that were located within the designed probe selection region, no other measure of probe set length or location was identified that was significantly different from INV probe sets and could explain the enhanced sensitivity to RNA degradation.

In comparisons of probe set level signal data from undegraded (RIN 9.5) RNA with RNA of progressively decreasing RNA integrity (RIN 8, 7, or 6), a substantial increase in the rate of detection of false positives was observed when RIN values are ≤ 7. Comparisons of samples with different RIN levels could occur in toxicogenomic studies where treatment conditions have induced a degree of damage and vehicle-treated control tissue is unaffected. Similar effects are possible in comparisons of results between single and multiple rounds of amplification. In one study, protocol method (one-cycle or two-cycle) was shown to have a bigger effect on signal variance than tissue type (breast vs. cervix) [3]

In our analysis of the effect of RNA integrity on assay sensitivity, probe set level signals from both "control" and "treated" samples were modeled from RNA with the same RIN. This design interrogates a decrease in sensitivity in studies where RNA integrity is similar for all samples but at lower than optimal levels because of tissue handling or RNA isolation method. The accuracy of true positive detection of *in silico *modeled changes of 1.5-, 2-, and 4-fold was reduced for RNA samples with RIN values ≤ 7. The effect was greatest for probe sets most sensitive to sample integrity and was less pronounced for probe sets unselected for an effect of RNA degradation on signal level.

## Conclusion

In this paper, we examined the effect of sample integrity on microarray performance through the use of samples with a progressive decrease in RNA quality that was indexed using a sensitive automated metric of RNA degradation (RIN). We identified a RIN threshold beyond which we observed a decrease in assay specificity and sensitivity. The effect on assay performance could be linked to a decrease in hybridization of target to probe sets that map more than 600 nt upstream of the transcription termini on their corresponding reference sequences.

## Methods

### Animal studies

Male Sprague Dawley rats were received from Harlan Laboratories (Frederick, MD) at 6 weeks of age and acclimated for 6 days. The rats received certified rodent diet #5002C (Purina Mills Inc.) *ad lib *and drinking water purified by reverse osmosis. The animals were on a 12 hr light/dark cycle and euthanasia was performed within 4 to 6 hr after the start of the light cycle. Animal care and procedures were approved by the Institutional Animal Care and Use Committee at the US FDA.

### Sample generation

After euthanasia by carbon dioxide inhalation, whole livers were removed and placed in Petri dishes containing sterile phosphate buffered saline (PBS). Livers were briefly rinsed with PBS to remove blood. Liver sections were prepared by removing a 2 cm square section from the left lateral lobe and further sectioning it into 12–16 equal pieces. Each time course study used a single liver lobe from a unique animal. For room temperature incubations, the sections were placed in RNAlater (Ambion, Austin, TX) at the end of each incubation period. Incubations at 37°C were performed in a water bath and terminated by addition of RNAlater. After the samples were incubated in RNAlater overnight, RNA was isolated using Qiagen Midi kits (Qiagen, Valencia, CA). To generate samples in which RNA was degraded after tissue thawing, liver sections were snap frozen in a dry ice/ethanol slurry and stored at -70°C. Random tissue sections were thawed at room temperature for various intervals. At the end of each incubation period, samples were homogenized in Qiagen RLT buffer and processed following the Qiagen Midi kit protocol. RNA and cRNA yields were quantitated on a NanoDrop ND-1000 spectrophotometer (NanoDrop Technologies, Wilmington, DE). All samples had 260/280 ratios ≥ 2.0. RNA integrity was characterized by measuring the 28s/18s rRNA ratio and RIN on an Agilent 2100 Bioanalyzer (Santa Clara, CA). RIN values for the zero-time point samples were ≥ 9.

cRNA was synthesized from 5 μg total RNA using Affymetrix standard protocols for cDNA synthesis and an IVT labeling kit from Enzo Life Sciences (Farmingdale, NY) for synthesis of biotin-labeled cRNA. Average cRNA lengths were calculated from standard curves generated from running the RNA 6000 Ladder (Ambion) on an Agilent 2100 Bioanalyzer and enabling smear analysis in the Agilent 2100 Expert software. A region was defined for each smear and the region start size was manually aligned with the vertical point of symmetry for each electropherogram. The region start sizes were automatically extrapolated from the standard curve data obtained from the 25 nt marker peak, and the first 5 RNA ladder fragment peaks in the RNA 6000 Nano Assay. The region end size and other values of the smear analysis table were not used to determine the median cRNA length.

To calculate statistically significant changes in RNA metrics between samples with RIN 9.5 and samples with RIN < 9.5, one-way ANOVA with a Dunnett's post test was performed using GraphPad Prism version 3.00 for Windows (GraphPad Software, San Diego, CA).

### Microarray experiments

Thirtytwo RNA samples from 7 independent time course studies of RNA degradation by F/T or 37°C incubation were labeled as described above and run on Affymetrix RAE230A arrays. 15 μg of fragmented cRNA was hybridized per array. Probe set signals were calculated using the Affymetrix MAS5 algorithm from files scaled to a target signal value of 500. The microarray data is available in EBI ArrayExpress under the accession number E-MEXP-1069 [23].

### Cluster analysis

Log_2 _signal ratios were calculated for probe sets in each non-control sample relative to the average zero-time control signal for each handling condition. Probe sets were identified that were either present in all control samples (n = 8) and had log_2 _ratios ≤ -1 in at least 50% of non-control 37°C or F/T samples or were present in all non-control samples within a handling condition set and had log_2 _ratios ≥ 1 in at least 30% of non-control 37°C or F/T samples. A total of 347 probe sets met these criteria within F/T or 37°C datasets. Hierarchical clustering was performed on this subset of probe sets for 28 non-control samples using Spotfire DecisionSite Functional Genomics software (Spotfire, Inc., Somerville, MA), Pearson correlation for the similarity measure, and the unweighted average clustering method. Gene Ontology classification was performed using DAVID [24].

### Probe set characterization

Probe sets that were significantly increased (INC), decreased (DEC), or unchanged (INV) by degradation were identified using the F/T RIN group replicates. Probe sets were identified as differentially expressed among the four RIN classes (9.5, 8, 7, and 6) by applying a multivariate permutation test (SAM) to provide a median false discovery rate of 10% using BRB-Array Tools Version 3.5.0 developed by Dr. Richard Simon and Amy Peng Lam. From this list of 574 probe sets, subsets of INC and DEC probe sets were identified that demonstrated a consistently increasing or decreasing monotonic trend in average signal between RIN 9.5, 8, 7 and 6 groups. From the probe sets that were not identified as significantly changed between RIN classes by SAM, a subset of INV probe sets were identified with signal values that did not significantly change as a function of RIN (signal coefficients of variation (CV) < 0.01 across all 12 F/T samples) and did not show a monotonic trend in average signal as a function of RIN.

Probe set location relative to the 5' and 3' ends of transcript reference sequences was calculated for DEC and INV probe sets that could be mapped to a single mRNA reference sequence (RefSeq) containing a terminal polyA sequence ≥ 10 nt. Mapping was defined as a 100% sequence match between the corresponding RefSeq and the first (No. 1) and last (No. 11) perfect match (PM) probes in the 11 probe series that comprises each probe set. The minimum contiguous sequence of a RefSeq that is targeted by all 11 probe pairs in a probe set is defined as the target sequence (TargetSeq).

Three distance metrics (3'-3' distance, 5'-3' distance, and 5'-5' distance) were determined for each probe set relative to its mapped position on the corresponding RefSeq. RefSeq lengths used for distance metrics excluded the length of the polyA sequence. The statistical significance of distance metric data between INV and DEC groups was calculated in unpaired two-tailed t-test comparisons using GraphPad Prism version 3.00 for Windows (GraphPad Software, San Diego, CA). The statistical significance of distance metric data between INV, DEC (5'-3' distance < 1000 nt), and DEC (5'-3' distance > 1000 nt) groups was calculated by applying a Tukey's post-test comparison of a one-way ANOVA using GraphPad software.

### Effect of RNA degradation on false positive and false negative rates

The effect of RNA degradation on the generation of false positives was analyzed by applying SAM at a median FDR of 0.1 in two-sample comparisons of control liver RNA that differed in RIN value using BRB-ArrayTools v3.5.0. Signals from the 3 F/T RIN 9.5 samples were compared to F/T RIN 8, 7, or 6 sample sets. For this analysis, signals were calculated using MAS5 or PLIER in ArrayAssist v4.0 (Stratagene, La Jolla, CA) for each comparison group of 6.

Probe sets with tissue-selective expression were identified from body map data generated on RAE230A arrays by applying a tissue-selective index cutoff of 5, as previously described [15]. Using this criterion, 292 probe sets with liver-selective signals (LS_ALL) and 188 probe sets with kidney-selective (KS) signals were identified. A subset of 30 LS probe sets (LS_DEC) was identified that were significantly changed by RNA degradation by applying SAM at a median FDR of 0.2 and that had decreasing monotonic trends in average log_2 _signal between the 4 F/T RIN groups (9.5 to 6). From the remaining LS probe sets that did not show a statistically significant difference in signal level between RIN groups using SAM, a set of 33 invariant probe sets (LS_INV) was identified that had log_2 _signals with CV ≤ 0.01 and a lack of monotonic trend in average signal across the 4 RIN groups ordered by decreasing RNA quality.

To model the effect of liver degradation on the diagnostic accuracy of detecting 1.5-fold changes in expression, the signal intensity from each F/T sample was used as the liver component to derive *in silico *signal intensities for the tissue-selective probe sets used as analytes in each of the two mixtures (Mix1 and Mix2) that comprise a mixed tissue RNA reference material using the formulas in [15]. "Biological" replicates of the modeled probe set signals in Mix1 and Mix2 were calculated for RIN group replicates through the use of a different, randomly assigned independent preparation of pooled brain, kidney, and testis RNA as the complex background (Batches 1–3 in [15]) for each replicate. To model the effect on diagnostic accuracy of 2-fold and 4-fold changes, the proportion of liver RNA signal in the mixtures was interchanged with the brain and testis RNA signal proportions, respectively. For each set of 3 RIN group replicates, a two sample t-test comparison of modeled Mix1 and Mix2 log_2 _signal values was performed to calculate a P value for each analyte. Subsets of LS_DEC, LS_INV, or LS_ALL analytes were used as true positives and the set of KS analytes were used as true negatives in ROC plots. The web-based program JROCFIT [25] was used to calculate the sensitivity at a 10% false positive rate from ROC plots of fitted data using the frequency of positives and negatives found in each of 36 exponentially spaced P value bins from 1 to 10^-7 ^(format 3). Where the data could not be fitted to a curve using JROCFIT, the sensitivity was interpolated from empirical data using the Weibull cumulative distribution function in Microsoft Excel.

## Abbreviations

RIN, RNA integrity number; F/T, freeze/thaw; nt, nucleotide; TargetSeq, target sequence; RefSeq, reference sequence; ROC, receiver operating characteristic; LS, liver-selective; KS, kidney-selective

## Competing interests

YT holds stock in Affymetrix Inc. KLT, BAR, and PSP receive collaborative research support from Affymetrix Inc.

## Authors' contributions

JR and KLT designed the study. BAR planned and conducted the laboratory experiments. KLT, PSP, and YT analyzed the data. The manuscript was drafted by KLT and edited by JDR. All authors approved the final manuscript.

## Supplementary Material

Additional file 1 Distance metrics for 3 probe sets that are sample quality controls on Affymetrix RAE230A arrays. This table contains distance metrics and average signal values for the endogenous control probe sets AFFX_Rat_GAPDH_5_at, AFFX_Rat_beta-actin_5_at, and AFFX_Rat_beta-actin_M_. Click here for file

Additional file 2 Lists of probe sets that were selectively altered by *ex vivo *incubation at 37°C. This file provides the probe set identifiers, gene symbols, UniGene identifiers, and gene names for probe sets increased or decreased by 37°C but not F/T incubation. Click here for file

Additional file 3 Distance metrics for individual probe sets. This file contains probe set identifiers, gene names, RefSeq identifiers, polyA lengths, TargetSeq lengths, RefSeq lengths, TargetSeq/RefSeq lengths, 3'-3' distance, 5'-3' distance, 5'-5' distance, and average log_2 _RIN 9.5 signal for probe sets classified by their sensitivity to RNA degradation (DEC or INV). Click here for file
