# Supplementary material for: Characterization of the effect of sample quality on high density oligonucleotide microarray data using progressively degraded rat liver RNA
Source: BMC Biotechnol. 2007 Sep 13;7:57. doi: 10.1186/1472-6750-7-57 (PMC2082023; doi:10.1186/1472-6750-7-57)
Supplement: Additional file 1 — Distance metrics for 3 probe sets that are sample quality controls on Affymetrix RAE230A arrays. This table contains distance metrics and average signal values for the endogenous control probe sets AFFX_Rat_GAPDH_5_at, AFFX_Rat_beta-actin_5_at, and AFFX_Rat_beta-actin_M_. [file 1472-6750-7-57-S1.pdf]

Additional Table 1:
Distance metrics for 3 probe sets that are sample quality controls on Affymetrix RAE230A arrays

**Distance Metric**	**GAPDH_5**	**Beta-actin_5**	**Beta-actin_M**
5'-3' Distance	1153	1173	783
3'-3' Distance	883	855	489
5'-5' Distance	124	93	483
RefSeq Length	1277	1266	1266
TargetSeq Length	271	319	295
TargetSeq/RefSeq	0.21	0.25	0.23
Avg RIN 9 Signal (log2)	11.81	11.96	13.04
