# Supplementary material for: Characterization of the effect of sample quality on high density oligonucleotide microarray data using progressively degraded rat liver RNA
Source: BMC Biotechnol. 2007 Sep 13;7:57. doi: 10.1186/1472-6750-7-57 (PMC2082023; doi:10.1186/1472-6750-7-57)
Supplement: Additional file 2 — Lists of probe sets that were selectively altered by ex vivo incubation at 37°C. This file provides the probe set identifiers, gene symbols, UniGene identifiers, and gene names for probe sets increased or decreased by 37°C but not F/T incubation. [file 1472-6750-7-57-S2.pdf]

Additional Table 2: Probe sets that were selectively altered by *ex vivo* incubation at 37°C

A. Increased by 37C incubation, not by F/T

Probe ID	Gene Symbol	Unigene ID	Gene Name
1368147_at	Dusp1	Rn.98260	dual specificity phosphatase 1
1368247_at	Hspa1a / Hspa1b	Rn.1950	heat shock 70kD protein 1A; heat shock 70kD protein 1B
1368321_at	Egr1	Rn.9096	early growth response 1
1369415_at	Bhlhb2	Rn.81055	basic helix-loop-helix domain containing, class B2
1370174_at	Myd116 / gadd34	Rn.2232	myeloid differentiation primary response gene 116
1371754_at	Slc25a25	Rn.17644	solute carrier family 25 (mitochondrial carrier, phosphate carrier), member 25
1386995_at	Btg2	Rn.27923	B-cell translocation gene 2, anti-proliferative
1387316_at	Cxcl1	Rn.10907	chemokine (C-X-C motif) ligand 1
1387870_at	Zfp36	Rn.82737	zinc finger protein 36
1389528_s_at	Jun	Rn.93714	v-jun sarcoma virus 17 oncogene homolog (avian)

B. Decreased by 37C incubation, not by F/T

Probe ID	Gene Symbol	Unigene ID	Gene Name
1367627_at	Gatm	Rn.17661	glycine amidinotransferase
1367679_at	Cd74	Rn.33804	CD74 antigen (invariant polpypeptide of MHC II antigen-associated)
1367850_at	LOC498276	Rn.131543	Fc gamma receptor II beta
1367948_a_at	Kdr	Rn.88869	kinase insert domain protein receptor
1367974_at	Anxa3	Rn.6589	annexin A3
1368558_s_at	Aif1	Rn.32080	allograft inflammatory factor 1
1368755_at	Clecsf13	Rn.9886	C-type lectin domain family 4, member f
1370883_at	RT1-Da	Rn.103146	RT1 class II, locus Da
1371079_at	Fcgr2b	Rn.33323	Fc receptor, IgG, low affinity IIb
1373025_at	C1qg_predicted	Rn.2393	complement component 1, q subcomponent, gamma polypeptide
1374334_at	Igha	Rn.102149	Immunoglobulin heavy chain (alpha polypeptide)
1376390_at	Ms4a11_predicted	Rn.101878	membrane-spanning 4-domains, subfamily A, member 11
1376652_at	C1qa_predicted	Rn.105647	complement component 1, q subcomponent, alpha polypeptide
1383169_at		Rn.40381	Transcribed locus, moderately similar to XP_580018.1
1385243_at	Maf	Rn.10726	V-maf musculoaponeurotic fibrosarcoma (avian) oncogene homolog
1387005_at	Ctss	Rn.11347	cathepsin S
1387794_at	Fcna	Rn.20051	ficolin A
1387902_a_at	LOC518 / LOC5183	Rn.126981	similar to Ig kappa chain V-V region K2 precursor
1389123_at	CCL6	Rn.7857	chemokine (C-C motif) ligand 6
1389659_at	LOC498690		similar to ctla-2-beta protein (141 AA) (predicted)
1390153_at		Rn.146145	Transcribed locus
1390798_at	Ptprc	Rn.90166	protein tyrosine phosphatase, receptor type, C
1398246_s_at	Fcgr3 / LOC498276	Rn.6050	Fc receptor, IgG, low affinity III; Fc gamma receptor II beta
