# Supplementary material for: Characterization of the effect of sample quality on high density oligonucleotide microarray data using progressively degraded rat liver RNA
Source: BMC Biotechnol. 2007 Sep 13;7:57. doi: 10.1186/1472-6750-7-57 (PMC2082023; doi:10.1186/1472-6750-7-57)
Supplement: Additional file 3 — Distance metrics for individual probe sets. This file contains probe set identifiers, gene names, RefSeq identifiers, polyA lengths, TargetSeq lengths, RefSeq lengths, TargetSeq/RefSeq lengths, 3'-3' distance, 5'-3' distance, 5'-5' distance, and average log2 RIN 9.5 signal for probe sets classified by their sensitivity to RNA degradation (DEC or INV). [file 1472-6750-7-57-S3.pdf]

Additional Table 3: Distance metrics for individual probe sets. This table contains probe set identifiers, gene names, RefSeq identifiers, polyA lengths, TargetSeq lengths, RefSeq lengths, TargetSeq/RefSeq lengths, 5′-3′ distance (distance from 5' end of the TargetSeq to the 3' end of the RefSeq), 3′-3′ distance (distance from 3' end of the TargetSeq to the 3' end of the RefSeq), 5′-5′ distance (distance from 5' end of the RefSeq to the 5' end of the TargetSeq), and average $\log_2$ RIN 9.5 signal for probe sets classified by their sensitivity to RNA degradation (DEC or INV). For distance measurement calculations, RefSeq lengths were corrected for differences in polyA length by subtracting this value. The DEC group is divided into two groups based on 5′-3′ distance (< or > 1000 nt).

Probe Set Sensitivity to RNA Degradation	Probe ID	Name	RefSeq Transcript ID	PolyA (pA) Correction	TargetSeq Length	RefSeq Length (-pA)	Covered Fraction	3'-3' Distance	5'-5' Distance	5'-3' Distance	Avg RIN 9.5 Signal (log2)
DEC (5'-3' distance < 1000 nt)	1367459_at	ADP-ribosylation factor 1	NM_022518	30	548	1759	0.31	142	1118	641	12.73
	1367651_at	cathepsin D	NM_134334	70	244	1990	0.12	377	1418	572	11.78
	1368079_at	pyruvate dehydrogenase kinase 1	NM_053826	30	426	1579	0.27	205	997	582	8.92
	1368921_a_at	CD44 antigen	NM_012924	43	547	3995	0.14	101	3396	599	7.96
	1369429_at	pyruvate dehydrogenase E1 alpha 2	NM_053994	12	403	2216	0.18	178	1684	532	5.46
	1369485_at	cytosolic acetyl-CoA hydrolase	NM_130747	19	549	1706	0.32	78	1128	578	10.37
	1369922_at	RDCR-0918-3 protein	NM_139255	12	302	1207	0.25	736	218	989	10.09
	1370215_at	complement component 1, q subcomponent, beta polypeptide	NM_019262	41	532	1095	0.49	412	200	895	10.33
	1371478_at	similar to RIKEN cDNA 1110008F13 (predicted)	NM_001013922	26	334	1019	0.33	91	643	376	11.39
	1371519_at	electron-transferring-flavoprotein dehydrogenase	NM_198742	26	394	2216	0.18	220	1651	565	11.16
	1371939_at	GPI-anchored membrane protein 1 (predicted)	NM_001012185	25	453	3288	0.14	152	2732	556	10.33
	1373058_at	similar to RIKEN cDNA 2010200I23	NM_001004248	25	529	1753	0.30	103	1170	583	11.89
	1375630_at	similar to NHP2-like protein 1 (High mobility group-like nuclear protein 2 homolog 1)	NM_212515	29	457	606	0.75	72	126	480	10.38
	1376073_at	Sel1 (suppressor of lin-12) 1 homolog (C. elegans)	NM_177933	27	461	3872	0.12	161	3298	574	10.49
	1376379_a_at	similar to RIKEN cDNA 2310004I24 gene (predicted)	NM_001009246	28	133	1261	0.11	556	621	640	7.82
	1387859_at	nitrogen fixation gene 1 (S. cerevisiae)	NM_053462	30	346	2008	0.17	256	1455	553	10.93
	1389062_at	RED protein	NM_001005537	37	517	1995	0.26	107	1420	575	9.30
	1389292_at	RAB18, member RAS oncogene family (predicted) /// similar to Rab18	NM_001012468	31	478	1745	0.27	149	1167	578	11.12
	1390687_at	pleckstrin (predicted)	NM_001025750	25	428	1646	0.26	72	1196	450	7.26
	1390717_at	similar to chromosome 20 open reading frame 155 (predicted)	NM_001014258	51	316	1870	0.17	700	903	967	8.63
	1393915_at	gene rich cluster, C3f gene (predicted)	NM_001012189	32	343	1903	0.18	562	1051	852	10.87
	1398863_at	guanine nucleotide binding protein, beta polypeptide 2	NM_031037	46	302	1624	0.19	688	684	940	10.80
	AFFX_Rat_beta-actin_M_at	actin, beta	NM_031144	30	295	1266	0.23	537	483	783	13.05
DEC (5'-3' distance > 1000 nt)	1367633_at	glutamine synthetase 1	NM_017073	30	453	4127	0.11	796	2927	1200	11.63
	1367855_at	scavenger receptor class B, member 1	NM_031541	22	506	2475	0.20	829	1189	1286	9.81
	1368218_at	ralA binding protein 1	NM_032067	20	566	3602	0.16	1189	1897	1705	6.37
	1368341_at	polymerase (DNA directed), beta	NM_017141	41	567	3257	0.17	2167	573	2684	7.30
	1368444_at	small glutamine-rich tetratricopeptide repeat (TPR)-containing, alpha	NM_022703	25	541	1971	0.27	825	655	1316	9.20
	1368808_at	CAP, adenylate cyclase-associated protein 1 (yeast)	NM_022383	25	519	2559	0.20	1129	960	1599	9.23
	1368847_at	RAB10, member RAS oncogene family	NM_017359	25	535	2713	0.20	1984	243	2470	9.72
	1368943_at	ribonuclease, RNase A family 4	NM_020082	25	436	1335	0.33	876	72	1263	13.64
	1368984_at	septin 2	NM_057148	34	471	3191	0.15	2026	745	2446	8.51
	1369077_at	N-acylsphingosine amidohydrolase 1	NM_053407	174	519	2265	0.23	1072	723	1542	9.03
	1369152_at	protein phospatase 3, regulatory subunit B, alpha isoform,type 1	NM_017309	29	553	2774	0.20	1923	347	2427	7.20
	1369234_at	solute carrier family 20, member 2	NM_017223	29	490	3626	0.14	1251	1934	1692	9.41
	1369558_at	inhibin beta C	NM_022614	41	509	1981	0.26	939	582	1399	9.71
	1369571_at	golgi phosphoprotein 3	NM_023977	26	534	2504	0.21	1488	531	1973	10.08
	1369621_s_at	FK506 binding protein 1a	NM_013102	25	274	1500	0.18	1147	128	1372	10.13
	1369640_at	gap junction membrane channel protein alpha 1	NM_012567	18	508	3130	0.16	2288	383	2747	7.50
	1369718_at	signal sequence receptor, gamma	NM_031120	19	490	2936	0.17	2429	66	2870	10.30
	1369791_at	putative chloride channel (similar to Mm Clcn4-2)	NM_022198	83	533	4332	0.12	1884	1964	2368	8.79
	1370005_at	cytochrome b5, outer mitochondrial membrane isoform	NM_030586	23	565	2722	0.21	1927	283	2439	10.47
	1370130_at	ras homolog gene family, member A	NM_057132	51	361	1832	0.20	1101	419	1413	10.42
	1370150_a_at	thyroid hormone responsive protein	NM_012703	68	81	1232	0.07	1186	14	1218	12.48
	1370501_at	ubiquitin-conjugating enzyme E2G 1 (UBC7 homolog, C. elegans)	NM_022690	60	555	1948	0.28	1043	403	1545	9.52
	1370725_a_at	glucose-6-phosphatase, catalytic	NM_013098	13	563	2224	0.25	415	803	1421	12.26
	1370772_a_at	hemochromatosis	NM_053301	21	346	3499	0.10	2468	734	2765	9.64
	1370951_at	ER transmembrane protein Dri 42	NM_138905	25	538	3080	0.17	949	1641	1439	7.73
	1371028_at	trans-golgi network protein 1	NM_138840	31	179	4713	0.04	1844	2739	1974	8.74
	1371161_at	protein phosphatase 1, regulatory (inhibitor) subunit 3B	NM_138912	25	454	4335	0.10	3377	553	3782	9.50
	1372437_at	S-phase kinase-associated protein 1A	NM_001007608	30	553	1217	0.45	504	209	1008	12.14

Probe Set Sensitivity to RNA Degradation	Probe ID	Name	RefSeq Transcript ID	PolyA (pA) Correction	TargetSeq Length	RefSeq Length (-pA)	Covered Fraction	3'-3' Distance	5'-5' Distance	5'-3' Distance	Avg RIN 9.5 Signal (log2)
DEC (5'-3' distance > 1000 nt)	1372506_at	proteaseome (prosome, macropain) 28 subunit, 3 (predicted)	NM_001011894	28	559	2614	0.21	1572	532	2082	9.47
(cont'd)	1374217_at	similar to chromosome 16 open reading frame 5 (predicted)	NM_001008360	28	413	2579	0.16	659	1556	1023	9.71
	1376727_at	similar to RIKEN cDNA 2310034L04 (predicted)	NM_001009712	28	310	1819	0.17	957	601	1218	10.25
	1383698_at	pyruvate dehydrogenase E1 alpha 1	NM_001004072	30	504	2807	0.18	1585	767	2040	9.88
	1386900_at	ribosome associated membrane protein 4	NM_030835	16	431	2426	0.18	1724	321	2105	11.36
	1386909_a_at	voltage-dependent anion channel 1	NM_031353	15	448	1803	0.25	873	531	1272	10.72
	1386918_a_at	opioid receptor, sigma 1	NM_030996	19	544	1571	0.35	600	476	1095	11.40
	1386958_at	thioredoxin reductase 1	NM_031614	17	509	3433	0.15	1211	1762	1671	10.33
	1387252_at	SEC14-like 2 (S. cerevisiae)	NM_053801	25	435	2589	0.17	1433	770	1819	10.33
	1387282_at	heat shock 22kDa protein 8	NM_053612	22	581	1773	0.33	887	354	1419	9.02
	1387358_at	ADP-ribosylation factor-like 1	NM_022385	31	479	1727	0.28	865	432	1295	10.20
	1387531_at	methionine sulfoxide reductase A	NM_053307	49	524	1299	0.40	712	112	1187	9.39
	1387665_at	betaine-homocysteine methyltransferase	NM_030850	20	521	1864	0.28	612	780	1084	13.22
	1387690_at	caspase 3	NM_012922	24	563	2460	0.23	1428	517	1943	9.11
	1387725_at	L-gulonolactone oxidase	NM_022220	33	559	2159	0.26	877	772	1387	12.95
	1387729_at	gamma-glutamyltransferase-like activity 1	NM_019235	44	548	2592	0.21	684	1409	1183	8.03
	1387757_at	liver regeneration p-53 related protein	NM_139189	28	302	2548	0.12	993	1302	1246	9.92
	1387776_at	transglutaminase 2, C polypeptide	NM_019386	19	480	3507	0.14	1238	1837	1670	9.98
	1387848_at	3-hydroxy-3-methylglutaryl-Coenzyme A reductase	NM_013134	14	484	4305	0.11	1273	2593	1712	8.12
	1387852_at	thyroid hormone responsive protein	NM_012703	68	389	1232	0.32	804	88	1144	10.58
	1387878_at	glutamate dehydrogenase 1	NM_012570	11	505	2863	0.18	713	1711	1152	13.39
	1388088_a_at	upstream transcription factor 2	NM_031139	10	496	2161	0.23	714	1000	1161	7.28
	1388748_at	lysosomal-associated protein transmembrane 4A	NM_199384	30	562	1285	0.44	528	244	1041	12.11
	1390040_at	brain and reproductive organ-expressed protein	NM_199270	30	409	1535	0.27	1150	25	1510	8.62
	1393480_at	protein phosphatase 1, regulatory (inhibitor) subunit 2	NM_138823	30	207	2232	0.09	1781	293	1939	6.58
	1394292_at	ribosomal protein, mitochondrial, L2	NM_001004235	25	296	1453	0.20	1131	75	1378	9.22
	1398273_at	ephrin A1	NM_053599	33	494	1452	0.34	847	160	1292	9.39
	1398281_at	thiopurine methyltransferase	NM_031329	20	553	4128	0.13	2230	1394	2734	7.04
	1398817_at	ADP-ribosylation factor 1	NM_022518	30	526	1759	0.30	944	339	1420	11.38
	1398822_at	GDP dissociation inhibitor 2	NM_017276	47	396	2183	0.18	917	919	1264	11.17
	1398823_at	translin-associated factor X	NM_022262	26	539	2264	0.24	935	839	1425	10.15
	1398824_at	coated vesicle membrane protein	NM_031722	30	508	1958	0.26	1322	177	1781	11.95
	1398825_at	RAB11B, member RAS oncogene family	NM_032617	18	517	1503	0.34	839	199	1304	10.13
	1398838_at	RAB7, member RAS oncogene family	NM_023950	22	464	2085	0.22	866	804	1281	10.69
	1399027_at	ras homolog gene family, member A	NM_057132	51	460	1832	0.25	599	822	1010	11.08
	1399043_at	capping protein (actin filament) muscle Z-line, alpha 2	NM_001009180	43	513	2308	0.22	570	1274	1034	10.33
	AFFX_Rat_beta-actin_5_at	actin, beta	NM_031144	30	319	1266	0.25	903	93	1173	11.82
	AFFX_Rat_GAPDH_5_at	glyceraldehyde-3-phosphate dehydrogenase	NM_017008	30	271	1277	0.21	931	124	1153	13.05
INV	1367461_at	coatomer protein complex, subunit beta 1	NM_080781	16	491	3332	0.15	141	2749	583	10.45
	1367640_at	ribosomal protein S12	NM_031709	118	328	545	0.60	183	83	462	12.81
	1367646_at	Cathepsin B	NM_022597	25	514	1942	0.26	89	1388	554	12.25
	1367671_at	proliferating cell nuclear antigen	NM_022381	30	424	1245	0.34	123	747	498	9.54
	1367679_at	CD74 antigen (invariant polpypeptide of major histocompatibility class II antigen-associated)	NM_013069	21	564	1199	0.47	63	621	578	11.37
	1367720_at	aminolevulinate, delta-, dehydratase	NM_012899	41	549	1237	0.44	90	647	590	11.91
	1367721_at	syndecan 4	NM_012649	10	530	2452	0.22	90	1882	570	11.85
	1367793_at	D-dopachrome tautomerase	NM_024131	18	514	610	0.84	105	40	570	13.30
	1367931_a_at	polypyrimidine tract binding protein 1	NM_022516	26	524	2697	0.19	88	2134	563	10.53
	1368205_at	complement factor I	NM_024157	13	509	2008	0.25	117	1431	577	13.49
	1368790_at	serine (or cysteine) proteinase inhibitor, clade A (alpha-1 antiproteinase, antitrypsin), member 10	NM_133617	26	555	1596	0.35	76	1014	582	12.19
	1368977_a_at	fractured callus expressed transcript 1	NM_053371	21	574	759	0.76	323	83	676	9.05
	1369930_at	proteasome (prosome, macropain) subunit, alpha type 6	NM_017283	176	512	961	0.53	123	375	586	11.71
	1370086_at	fibrinogen, gamma polypeptide	NM_012559	27	466	1527	0.31	106	1004	523	14.33

Probe Set Sensitivity to RNA Degradation	Probe ID	Name	RefSeq Transcript ID	PolyA (pA) Correction	TargetSeq Length	RefSeq Length (-pA)	Covered Fraction	3'-3' Distance	5'-5' Distance	5'-3' Distance	Avg RIN 9.5 Signal (log2)
INV	1370166_at	syndecan 2	NM_013082	25	410	2949	0.14	1280	1308	1641	12.22
(cont'd)	1370167_at	syndecan 2	NM_013082	25	512	2949	0.17	2230	256	2693	12.61
	1370242_at	ribosomal protein S23	NM_078617	29	357	514	0.69	157	49	465	13.27
	1370244_at	cathepsin L	NM_013156	10	481	1376	0.35	224	720	656	12.44
	1370253_at	ribosomal protein L22	NM_031104	13	361	452	0.80	75	65	387	12.27
	1370881_at	thiosulfate sulfurtransferase	NM_012808	26	467	1093	0.43	119	556	537	12.63
	1371249_at	X-box binding protein 1	NM_001004210	30	472	1821	0.26	99	1299	522	11.98
	1371251_at	galactose-1-phosphate uridyl transferase (predicted)	NM_001013089	68	472	1307	0.36	103	781	526	11.27
	1371307_at	ribosomal protein, large, P1	NM_001007604	23	448	499	0.90	78	22	477	13.22
	1371312_at	coiled-coil-helix-coiled-coil-helix domain containing 2 (predicted)	NM_001015019	30	500	663	0.75	124	88	575	12.64
	1371352_at	high mobility group protein 17	NM_001025624	47	412	1193	0.35	207	622	571	11.56
	1371465_at	cortactin	NM_021868	33	528	2918	0.18	95	2344	574	9.26
	1371528_at	FK506 binding protein 8 (predicted)	NM_001037180	14	578	1665	0.35	68	1068	597	12.09
	1371615_at	diacylglycerol O-acyltransferase homolog 2 (mouse)	NM_001012345	26	501	2294	0.22	85	1757	537	12.55
	1371634_at	similar to RIKEN cDNA 1810020E01 (predicted)	NM_001011557	28	411	2483	0.17	1897	224	2259	9.68
	1371688_at	translocating chain-associating membrane protein	NM_001007701	24	529	2754	0.19	109	2165	589	12.88
	1371780_at	KDEL (Lys-Asp-Glu-Leu) endoplasmic reticulum protein retention receptor 2 (predicted)	NM_001013122	30	491	1779	0.28	84	1253	526	11.31
	1372077_at	serine/threonine kinase receptor associated protein (predicted)	NM_001011969	25	477	1730	0.28	83	1219	511	10.75
	1372372_at	similar to Ab2-225 (predicted)	NM_001008770	29	460	1115	0.41	69	635	480	12.11
	1372612_at	dynein light chain-2	NM_080697	47	478	2323	0.21	61	1833	490	11.16
	1372672_at	quinolinate phosphoribosyltransferase (predicted)	NM_001009646	38	564	1187	0.48	49	623	564	11.29
	1373048_at	ARP10 actin-related protein 10 homolog (S. cerevisiae) (predicted)	NM_001009602	27	424	1430	0.30	200	855	575	9.69
	1373059_at	ankyrin repeat domain 13 (predicted)	NM_001012148	31	545	3641	0.15	452	2693	948	10.14
	1373452_at	RNA terminal phosphate cyclase-like 1 (predicted)	NM_001013152	28	557	1331	0.42	80	743	588	12.14
	1374061_at	similar to RIKEN cDNA 1110055L24 (predicted)	NM_001013916	24	199	1210	0.16	166	894	316	11.75
	1375440_at	peptidylprolyl isomerase (cyclophilin)-like 2 (predicted)	NM_001017383	25	542	1776	0.31	85	1198	578	8.86
	1376086_at	similar to RIKEN cDNA 1810047C23 (predicted)	NM_001014142	26	510	1513	0.34	114	938	575	9.78
	1377060_at	methylcrotonoyl-Coenzyme A carboxylase 2 (beta) (predicted)	NM_001012177	78	375	2142	0.18	93	1723	419	10.82
	1386925_at	actin related protein 2/3 complex, subunit 1B	NM_019289	36	488	1500	0.33	126	935	565	9.71
	1387240_at	retinol dehydrogenase 7	NM_133543	26	577	1148	0.50	52	569	579	12.66
	1388634_at	phosphoglucomutase 1	NM_017033	27	421	2280	0.18	88	1820	460	11.10
	1388681_at	SAR1a gene homolog 2 (S. cerevisiae) (predicted)	NM_001009622	33	532	1157	0.46	94	580	577	11.05
	1388948_at	START domain containing 10 (predicted)	NM_001013069	84	491	1250	0.39	63	745	505	12.90
	1388997_at	ADP-ribosylation factor 3	NM_080904	30	558	3285	0.17	70	2706	579	10.32
	1389480_at	RWD domain containing 4A	NM_001034994	92	490	2891	0.17	112	2338	553	10.52
	1389575_at	similar to sid2057p (predicted)	NM_001013898	27	363	1432	0.25	241	877	555	10.35
	1390125_at	transmembrane 9 superfamily member 1 (predicted)	NM_001012155	33	405	2408	0.17	87	1965	443	10.39
	1398294_at	actinin, alpha 1	NM_031005	26	484	3670	0.13	700	2535	1135	8.68
	1398324_at	similar to 60S ribosomal protein L18a	NM_212510	29	532	616	0.86	84	49	567	12.76
	1398326_at	similar to Nur77 downstream protein 2	NM_001007008	22	516	618	0.84	96	55	563	13.23
	1398339_at	zinc finger protein 162	NM_058210	26	361	2649	0.14	124	2213	436	10.43
	1398753_at	aldo-keto reductase family 1, member A1	NM_031000	30	541	1407	0.38	77	838	569	12.34
	1398755_at	ATPase, H+ transporting, V0 subunit C	NM_130823	30	404	1156	0.35	216	586	570	12.04
	1398768_at	retinoblastoma binding protein 7	NM_031816	25	543	1922	0.28	70	1358	564	10.91
	1398796_at	transmembrane trafficking protein 21	NM_053467	28	455	1286	0.35	161	719	567	11.11
	1398800_at	tyrosine 3-monooxygenase/tryptophan 5-monooxygenase activation protein, beta polypeptide	NM_019377	60	477	2696	0.18	129	2139	557	10.83
	1398880_at	RNA polymerase II transcriptional coactivator	NM_001009618	26	482	756	0.64	70	253	503	11.94
